# Individual Differences in the Attentional Blink: The Temporal Profile of Blinkers and Non-Blinkers

**DOI:** 10.1371/journal.pone.0066185

**Published:** 2013-06-03

**Authors:** Charlotte Willems, Stefan M. Wierda, Eva van Viegen, Sander Martens

**Affiliations:** 1 Neuroimaging Center, University of Groningen, Groningen, The Netherlands; 2 Department of Neuroscience, University Medical Center Groningen, Groningen, The Netherlands; 3 Department of Artificial Intelligence, University of Groningen, Groningen, The Netherlands; University of California, Davis, United States of America

## Abstract

**Background:**

When two targets are presented in close temporal succession, the majority of people frequently fail to report the second target. This phenomenon, known as the ‘attentional blink’ (AB), has been a major topic in attention research for the past twenty years because it is informative about the rate at which stimuli can be encoded into consciously accessible representations. An aspect of the AB that has long been ignored, however, is individual differences.

**Methodology/Principal Findings:**

Here we compare a group of blinkers (who show an AB) and non-blinkers (who show little or no AB), and investigate the boundary conditions of the non-blinkers' remarkable ability. Second, we directly test the properties of temporal selection by analysing response errors, allowing us to uncover individual differences in suppression, delay, and diffusion of selective attention across time. Thirdly, we test the hypothesis that information concerning temporal order is compromised when an AB is somehow avoided. Surprisingly, compared to earlier studies, only a modest amount of suppression was found for blinkers. Non-blinkers showed no suppression, were more precise in selecting the second target, and made less order reversals than blinkers did. In contrast, non-blinkers made relatively more intrusions and showed a selection delay when the second target immediately followed the first target (at lag 1).

**Conclusion/Significance:**

The findings shed new light on the mechanisms that may underlie individual differences in selective attention. The notable ability of non-blinkers to accurately perceive targets presented in close temporal succession might be due to a relatively faster and more precise target selection process compared to large blinkers.

## Introduction

Restrictions to concurrent attention and awareness are revealed by the interference that commonly results when two sensory inputs must be identified closely in time. For instance, the majority of people typically fail to report the second of two targets when presented in close temporal succession (200–500 ms) amongst a sequential stream of distractors, a phenomenon known as the attentional blink (AB) [1], [2].

In the past two decades, the AB has been a major topic in attention research because it is informative about the rate at which stimuli can be encoded into consciously accessible representations. Although the effect is robust and can be obtained under a variety of task conditions [1], large individual differences exist in the magnitude of the effect [3]–[5]. Such differences have long been considered as irrelevant noise, until we demonstrated that for some individuals (referred to as ‘non-blinkers’) the AB can be completely absent [3]. Given that there is currently much debate about the cause of the AB (see [1], [6] for recent reviews), several subsequent studies have focused on individual differences in AB magnitude in an attempt to shed new light on the underlying mechanism of the AB [1], [3], [7]–[25].

Representing the extreme end on a continuum of individual AB magnitudes, non-blinkers continue to show little or no AB when identification of targets is made more difficult by either increasing the overall rate of stimulus presentation [3] or specifically reducing the duration of the targets [16], [18], [20]. In comparison to regular ‘blinkers’ (individuals who do show an AB), it has been found that non-blinkers neither seem to differ in short-term memory capacity, working memory capacity, nor in general intelligence level [17] (but see [8], [10], which do report a relation between WM capacity and AB magnitude).

In contrast, however, EEG measurements have revealed differences in frontal and parietal brain activity, reflecting differences in target processing [3]. In particular, more target-related activity was found over the ventrolateral prefrontal cortex (assumed to play a role in a wide range of cognitive processes, including the selection of non-spatial information), whereas blinkers showed more distractor-related prefrontal activity. Regardless of the time interval between the targets, non-blinkers were also found to be quicker in consolidating the identity of targets than blinkers, showing earlier peak latencies of the P3 ERP components—associated with the updating of working memory (WM)—induced by successfully identified targets [3]. In line with this result, evidence was recently found that the magnitude of the AB is related to striatal dopamine functioning, which is associated with regulating the threshold for WM updating [26]. Taken together, these findings suggest that non-blinkers are more efficient in distinguishing targets from distractors at a relatively early processing stage. Indeed, behavioural studies have provided converging evidence showing that non-blinkers are better in ignoring distractors than blinkers are [14], [18], [21].

It must be noted though that this early selection seems to be specific for alphanumeric, visual targets. AB magnitude was found to be similar for blinkers and non-blinkers when using pictures rather than alphanumeric stimuli [16]. Also when using auditory alphanumeric stimuli, non-blinkers showed a substantial AB effect, although overall performance was still better than that of blinkers [18].

It was therefore suggested that in an alphanumeric AB task non-blinkers might take advantage of overlearned category-level features to select targets prior to full identification, allowing them to mostly ignore distractors and to avoid an AB. Indeed, an ERP study subsequently showed that when alphanumeric category information was unavailable (only letters were presented) and target selection could only be based on information that is processed relatively late (rotation), non-blinkers again showed a substantial AB effect [20]. Delayed target-related occipito-parietal activity as well as increased distractor-related prefrontal brain activity was observed. Also, when alphanumeric category information was not available, the difference in P3 peak latency between the two groups disappeared. However, non-blinkers continued to outperform blinkers across all conditions by showing a smaller AB, suggesting that early selection processes based on category information alone cannot fully explain the observed differences between the two groups.

Nevertheless, it has been suggested that a major source of individual variability in AB magnitude must lie in processes of selective attention that are involved in determining which objects are selected for further processing and memory consolidation [3], [16]–[18], [20], [21]. In this regard, the insights derived from studies examining individual differences in the AB converge with recent ideas regarding the source of the AB. Whereas the earliest studies claimed that the AB is the result of capacity limitations [27],[28], alternatively, the AB is lately often regarded as a problem to time or control attention [1], [6], [23], [29], [30]. This shift in the theoretical landscape was motivated by a number of key findings. For instance, it was found that people are capable of reporting an undisrupted stream of letters, but typically fail when required to report only a subset of this stream, as reflected in the AB task [31], [32]. Furthermore, it has been found that the AB is attenuated when participants perform a second task concurrently with the primary AB task [23], [33]–[35]. Together, these studies provide evidence against theories assuming resource depletion, since according to these limited-capacity theories an additional task load should increase rather than decrease the magnitude of the AB. Given these findings, the temporal selection mechanism seems important for explaining the AB, although it must be noted that recent findings also suggest a role for capacity limitations [36]–[39].

The aim of the present study was to further investigate this temporal selection mechanism by contrasting the performance of blinkers and non-blinkers. In the abovementioned studies, non-blinkers showed an AB when visual target selection was based on a target-defining feature that was processed relatively late, such as rotation [20] or semantic category [16]. To test the generality of this finding, an AB experiment was set up that featured only letter stimuli with targets defined by colour, a stimulus feature that is available relatively early [40]–[42]. This way, early target selection should be possible, and non-blinkers should still be able to avoid an AB on the majority of trials. However, if their temporal selection ability specifically relies on the presence of alphanumeric category information—which is unavailable—the occurrence of an AB is to be expected.

To study the temporal dynamics of attention in more detail, another important goal of the current study was to investigate the temporal profile of non-blinkers and blinkers using three measures of temporal selection, namely ‘suppression’, ‘delay’, and ‘diffusion’, originally proposed by Vul et al. [43] and Chun [44]. Since each stimulus letter was presented only once within each stream, the serial position of any reported letter was known, thus allowing us to highlight and contrast these three dimensions of target selection in blinkers and non-blinkers [43]. Following Vul and colleagues, if a response consists of a letter that does not correspond with any of the letters presented within a certain temporal window around a target, we assume that the relevant information was likely to be suppressed (‘suppression’). If a response corresponds with a letter that was presented after a target, it can be inferred that temporal target selection was delayed (‘delay’). Finally, if distractors strongly interfere with the processing of targets, selection will be less precise, reflected in selection errors that are temporally more distant from the target (‘diffusion’). Vul et al. [43] found that the temporal selection process was suppressed, delayed, and diffused during the AB.

Both the concepts of suppression and delay have previously been associated with the AB. Regarding suppression, many studies emphasized its important role during the AB [14], [21], [43]–[54]. In EEG studies, suppression is reflected in the P3 component that is absent or strongly attenuated during the AB [53], and also the n2pc (associated with the allocation of attention) is known to be affected [55]–[57]. Similarly there is quite some evidence supporting the idea that attentional selection is delayed during the AB, provided by behavioural studies [27], [43], [44], [50], [58]–[60] and EEG studies [3], [53], [61], where the latter have revealed that when the second target was reported correctly at short time intervals, the P3 component was delayed in comparison to longer intervals.

Combined with our previous findings on individual differences in the AB, we predicted that non-blinkers would continue to outperform the blinkers, and would show less suppression, delay, and diffusion. Interestingly however, although many papers suggested that information processing is suppressed during the attentional blink (e.g., [43], [51], [53], [54]), a number of papers have claimed that the AB is due to a failure to suppress distractor stimuli [14], [46], [47], [52], which implies that we should find the opposite effect; individuals with little or no AB should show relatively strong suppression, whereas individuals with a large AB should show relatively little suppression.

A final prediction concerning non-blinker performance comes from a simulation study suggesting the AB to reflect a cognitive strategy of enforcing an episodic distinction between successive stimuli [29]. When the occurrence of an AB is somehow avoided, information concerning temporal order and the correct binding of features into targets might be compromised [1]. In other words, non-blinkers might lack the episodic distinction between successive stimuli, and subsequently make more order reversals (i.e., reporting the second target before the first target) than blinkers do. If however, non-blinkers are generally quicker to select and consolidate targets (see e.g., [3]), one would expect to find fewer order reversals in non-blinkers than in blinkers. A final aim was thus to test these latter predictions.

In summary, we tested whether non-blinkers can avoid an AB when targets are to be selected on the basis of colour rather than alphanumeric category information. Second, we tested whether non-blinkers show less suppression, delay, and diffusion than blinkers do. And third, we investigated whether avoiding an AB comes at a cost, reflected in non-blinkers making relatively more order reversals.

## Methods

Experiment 1a consisted of an AB task with alphanumeric stimuli, requiring detection and identification of two target letters presented in a rapid serial visual presentation (RSVP) stream of 16 distractor digits. Participants were tested for the presence or absence of a sizeable AB, with the purpose of forming separate groups of consistent blinkers and non-blinkers for inclusion in Experiment 1b. Experiment 1b contained only letter stimuli, targets were defined by colour, and its goal was to test the temporal profile of blinkers and non-blinkers in terms of suppression, delay, and diffusion. The purpose of Experiment 2 was to replicate the findings in a larger sample of participants.

### Experiment 1a

In Experiment 1a, participants performed an AB task requiring the identification of two letter targets amongst a sequential stream of digit distractors. The purpose of this experiment was to test selected participants for the presence or absence of a sizeable AB in a classical alphanumeric AB task. In addition, we aimed to systematically study possible differences between blinkers and non-blinkers in terms of order reversals.

#### Participants

Twenty-nine volunteers (16 women; aged 20–31, mean  = 25.0) recruited from the University of Groningen community participated in the experiment, had normal or corrected-to-normal visual acuity, normal hearing, and no history of neurological problems. One participant was excluded due to RSI problems. Thirteen participants were included because they had shown little or no AB in previous studies in our laboratory, and were therefore regarded as potential non-blinkers. The other 15 participants had previously shown a regular to large AB, and were therefore regarded as potential blinkers. The Neuroimaging Center Institutional Review Board approved the experimental protocol and each participant signed a written consent prior to the experiment. All volunteers participated in both Experiment 1a and 1b in a single session, and received payment of € 7 in total.

#### Stimuli and apparatus

The generation of stimuli and the collection of responses were controlled by using E-prime 1.2 software running under Windows XP. Target stimuli consisted of uppercase consonant letters excluding ‘Q’, ‘V’, and ‘Y’. Distractor stimuli consisted of digits (2 to 9). All stimuli were centrally presented in black (2 cd/m^2^) on a white background (88 cd/m^2^) in uppercase 14-point Monaco font on a 19-inch CRT monitor with a 100-Hz refresh rate. Viewing distance was approximately 50 cm.

#### Procedure

Each trial began with a message at the bottom of the screen, prompting participants to press the space bar to initiate the trial. When the space bar was pressed, the message disappeared immediately and a central fixation cross appeared. It remained on the screen for 100 ms, followed by the RSVP stream consisting of 18 items (i.e., 2 targets and 16 distractors).

All stimuli were presented for 80 ms without inter stimulus interval. The first target (T1) was always presented as the sixth item in the stream. The second target (T2) was the first, second, third, or eighth item following T1, and was thus presented at lag 1, 2, 3, or 8, respectively. In other words, the stimulus onset asynchrony (SOA) between the targets randomly varied from 80, 160, 240, to 640 ms. Each lag was presented equally often. Target letters were pseudo-randomly selected with the constraint that T1 and T2 were always different letters. Digit distractors were pseudo-randomly selected with the constraint that no single digit was presented twice in succession.

After the presentation of the stimulus stream, participants were prompted by a message at the bottom of the screen to indicate the letters they had seen by using the corresponding keys on the computer keyboard. Participants were instructed to take sufficient time in making their responses to ensure that typing errors were avoided. Participants were encouraged to type in their responses in the order in which the letters had been presented, but responses were accepted and counted correct in either order. Participants were instructed to guess if they had not seen the targets.

The experiment contained one practice block of 24 trials and two testing blocks of 144 trials each, and took approximately 30 minutes to complete. After the first testing block, participants were allowed to take a short break. At the end of the experiment, participants took another short break before continuing with Experiment 1b.

### Experiment 1b

The purpose of Experiment 1b was twofold. First, we wanted to test whether non-blinkers continue to show little or no AB when targets are defined by colour rather than alphanumeric category. To that end, all stimuli consisted of letters, with targets presented in red, and distractors in black. Second, following [43], we directly tested the properties of temporal selection by analysing the distribution of reported letters, allowing us to study the suppression, delay, and diffusion of selective attention across time in blinkers and non-blinkers.

#### Participants

All participants of Experiment 1a volunteered to participate in Experiment 1b. Participants were assigned to the same groups of blinkers and non-blinkers as in Experiment 1a. Note that the individuals who consistently show no AB in an alphanumeric AB task as demonstrated in Experiment 1a (i.e., non-blinkers) might show an AB under the experimental conditions of Experiment 1b. To consistently refer to these individuals in Experiments 1a and 1b, we will continue to label them as ‘non-blinkers’, keeping in line with the literature on non-blinkers [18], [20].

#### Stimuli and apparatus

The same stimuli and apparatus were used as in Experiment 1a, except that all stimuli consisted of consonant letters. Again ‘V’, ‘Q’, ‘Y’ were excluded. Targets were presented in red, whereas distractors were presented in black.

#### Procedure

The procedure was the same as in Experiment 1a, except that all stimuli were presented for 120 ms, such that a similar level of difficulty was obtained as in Experiment 1a. Furthermore, the RSVP consisted of 16 stimuli, and T1 was always presented as the fifth item in the stream. Experiment 1b took approximately 35 minutes to complete.

### Experiment 2

The aim of Experiment 2 was to strengthen the results found in Experiment 1b by replicating the results in a larger sample of participants, enabling us to study a wider range of individual differences.

#### Participants

A total of 132 volunteers (98 women) recruited from the University of Groningen participated in the experiment in return for course credits. Unfortunately, due to technical problems, the age related information of the participants was lost for this experiment. However, because participants were selected from a similar pool of participants as in Experiment 1, it can be assumed that the average age of the participants in both experiments was equivalent. They had normal or corrected-to-normal visual acuity, normal hearing, and no history of neurological problems. The Neuroimaging Center Institutional Review Board approved the experimental protocol and each participant signed a written consent prior to the experiment.

#### Stimuli and apparatus

The stimuli and apparatus were the same as in Experiment 1b.

#### Procedure

The procedure was similar to that in Experiment 1b. The experiment consisted of one practice block of 14 trials and three testing blocks of 96 trials each. Participants were allowed to take a short break between blocks. They completed the experiment in approximately 45 minutes.

## Results and Discussion

When appropriate, Greenhouse-Geisser-corrected *p* values are reported (ε<0.75). In addition, a Bonferroni-correction was applied when independent t-tests were performed serving as post-hoc test.

### Experiment 1a

To assure that participants were assigned to the appropriate group, AB magnitude was first computed for each individual by calculating the percentage decline in T2 accuracy at lags 2 and 3 relative to T1 accuracy across lags. Following previous non-blinker studies [17], [62], [63], the AB magnitude was calculated as a function of T1 accuracy by using the following formula: 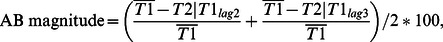
where 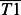 is the mean accuracy of T1, and T2|T1_lag_ is the mean accuracy of T2 at a specific lag given that T1 was correctly reported. We used this particular method to assure that individuals with a high T1 accuracy, but overall low T2 accuracy were not erroneously classified as non-blinkers. However, alternative ways to calculate AB magnitude, for instance by relating T2 accuracy at lags 2 and 3 to T2 accuracy at lag 8 produced comparable results. Mean AB magnitude was 8.7% for the non-blinkers, ranging from 2.5% to 15.3%, suggesting that each individual within this group indeed showed little or no AB. For the blinkers, mean AB magnitude was 32.6%, ranging from 17.0% to 50.6%, suggesting that they showed a moderate to large AB.

**Figure 1:**
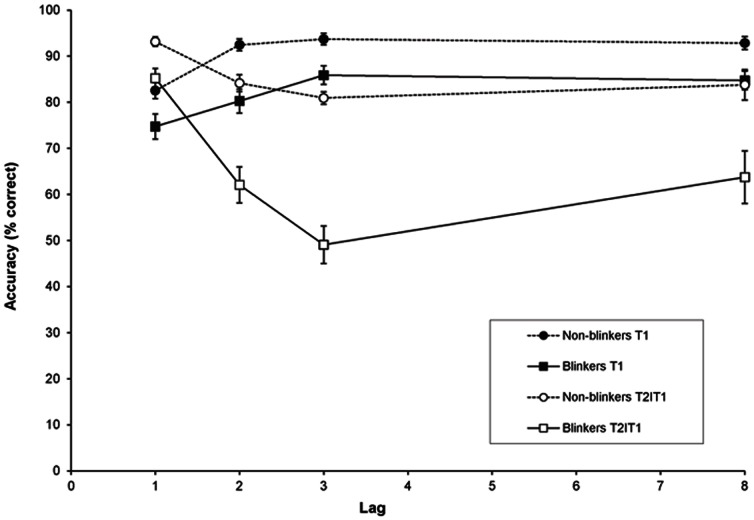
Target accuracy in Experiment 1a. Mean percentage correct report of T1 (black symbols) and T2 given correct report of T1 (white symbols) as a function of lag, for non-blinkers (circles) and blinkers (squares). Error bars reflect standard error of the mean.

Figure 1 shows target accuracy as a function of the interval between the two targets (lag), for non-blinkers (circle symbols) and blinkers (square symbols). A repeated measures analysis of variance (RM-ANOVA) of T1 accuracy with group (non-blinkers and blinkers) as a between-subjects factor and lag (1, 2, 3, and 8) as a within-subjects factor revealed a significant effect of group, *F*(1,26) = 13.49, *MSE*  = 166.64, *p* = .001, *η* ^2^ *_p_* = .34, reflecting mean accuracy to be higher for non-blinkers (90.4%) than for blinkers (81.4%). In addition, a main effect of lag was found, *F*(2.17, 56.49) = 33.27, *MSE*  = 29.43, *p*<.001, *η* ^2^ *_p_* = .56, such that performance at lag 1 was relatively low. The Group × Lag interaction was not significant (*p* = .23).

A RM-ANOVA of T2 performance given correct report of T1 (T2|T1) with group as a between-subjects factor and lag as a within-subjects factor revealed a significant effect of group, *F*(1,26) = 28.75, *MSE*  = 406.53, *p*<.001, *η* ^2^ *_p_* = .53; lag, *F*(3, 78) = 33.63, *MSE*  = 84.49, *p*<.001, *η* ^2^ *_p_* = .56; and a significant Group × Lag interaction, *F*(3, 78) = 7.94, *MSE*  = 84.49, *p* = .001, *η* ^2^ *_p_* = .23. Separate analyses in which lag 1 was excluded revealed that non-blinkers did not show a significant AB (*p* = .38), whereas blinkers did, *F*(2,28) = 8.11, *MSE*  = 119.22, *p* = .002, *η* ^2^ *_p_* = .37.

#### Order reversals

We calculated the relative percentage of order reversals over the trials where T1 and T2 were both correctly reported, providing a measure of order reversals that is irrespective of individual differences in identification accuracy. Interestingly, there was a significant effect of group, *F*(1,26) = 5.96, *MSE*  = 152.26, *p* = .022, *η* ^2^ *_p_* = .19, such that non-blinkers showed relatively fewer order reversals than blinkers did (11.1% vs. 16.8%, respectively). In addition, we found an effect of lag, *F*(3, 78) = 83.99, *MSE*  = 51.77, *p*<.001, *η* ^2^ *_p_* = .76, as the number of order reversals decreased as a function of lag (30.8%, 14.8%, 10.0%, and .9% at lags 1, 2, 3, and 8, respectively). Also a marginally significant Group × Lag interaction was found, *F*(3, 78) = 2.71, *MSE*  = 51.77, *p* = .051, *η* ^2^ *_p_* = .09, such that particularly at lags 2 and 3, non-blinkers seemed to show fewer order reversals than blinkers did.

### Experiment 1b

**Figure 2:**
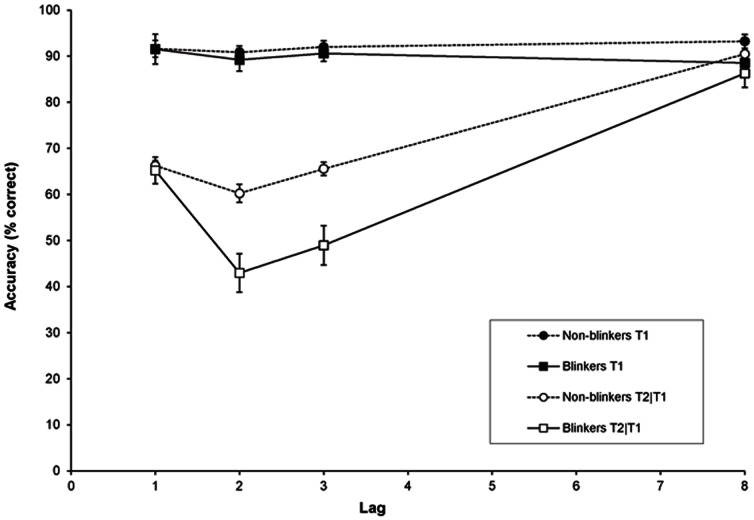
Target accuracy in Experiment 1b. Mean percentage correct report of T1 and T2 given correct report of T1 as a function of lag, for non-blinkers and blinkers. Error bars reflect standard error of the mean.

Figure 2 shows target accuracy as a function of lag, for non-blinkers and blinkers. Mean T1 accuracy was 90.0% for the blinkers and 91.9% for the non-blinkers. A RM-ANOVA of T1 performance revealed no significant effects (*p*s>.10).

A RM-ANOVA of T2|T1 revealed a significant effect of group, *F*(1,26) = 8.98, *MSE*  = 296.76, *p* = .006, *η* ^2^ *_p_* = .26; lag, *F*(3, 78) = 73.40, *MSE*  = 99.27, *p*<.001, *η* ^2^ *_p_* = .74; and a significant Group × Lag interaction, *F*(3, 78) = 4.93, *MSE*  = .9.27, *p* = .007, *η* ^2^ *_p_* = .16. Mean AB magnitude was 31.6% for non-blinkers and 49.0% for blinkers (*t*(26) = 3.53, *SE* = 4.95, *p* = .002). These findings suggest that both the blinkers as well as the non-blinkers showed a sizeable AB, but that it was substantially smaller in the non-blinkers than in the blinkers.

A positive Pearson product-moment correlation was found between individual AB magnitudes in Experiments 1a and 1b, *r* = .42, *p* = .027. A similar correlation was found for T2|T1 performance, *r* = .44, *p* = .019, but not for T1 performance (*p* = .14). These findings suggest that although AB magnitude was generally larger in Experiment 1b than in Experiment 1a, individuals with a relatively small or large AB in Experiment 1a continued to show a relatively small or large AB in Experiment 1b, respectively.

#### Suppression

We estimated the efficacy of selection (*A*) as the proportion of trials during which an item was reported from a 7-item window around the target (spanning three items before to three items after the target) as follows: 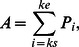
where *P_i_* is the probability (i.e., empirical frequency) of reporting an item from serial position *i* relative to the target position (*i* = 0), and *ks* and *ke* are the lower and upper bounds, respectively, of the window used to compute the measure (in this case, −3 and 3, respectively). Thus, we calculated how frequent each participant reported a letter from the 7-item window surrounding T1 or T2 to indicate the availability of the distractors around the target. In contrast to the previous analyses, order reversals were counted as incorrect, because for these and the following analyses we were interested in the exact serial location of the reported letters.

**Figure 3:**
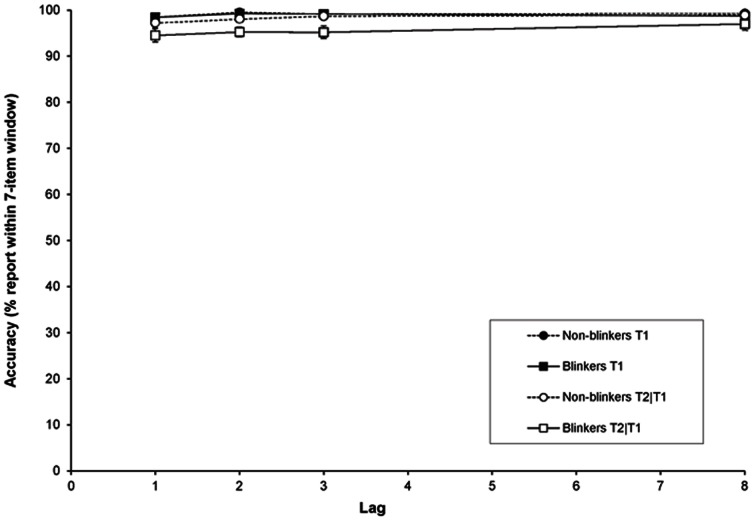
Suppression in Experiment 1b. Suppression of the temporal selection process expressed as the accuracy of reporting an item within the 7-item window around a given target as a function of lag, for blinkers and non-blinkers.

As shown in Figure 3, performance within the 7-item window was close to or at ceiling for both blinkers and non-blinkers. Given that 17 different letters could be presented within the stream, the chance to randomly select a letter within the 7-item window was 7/17 (i.e., 42%). A paired t-test revealed that the accuracy of reporting an item within the 7-item window differed significantly from the level of chance, *t*(27) = 60.0, *SE* = .9, *p*<.001; *t*(27) = 74.29, *SE* = .74, *p*<.001; *t*(27) = 61.0, *SE* = .91, *p*<.001; *t*(27) = 68.5, *SE* = .82, *p*<.001 for lags 1, 2, 3, and 8, respectively. A RM-ANOVA of T1 showed an effect of lag, *F*(3, 78) = 4.1, *MSE*  = 1.09, *p* = .009, *η* ^2^ *_p_*  = .14, but both the Group x Lag interaction (*p* = .66), as the effect of group (*p* = .62) were non-significant.

**Figure 4:**
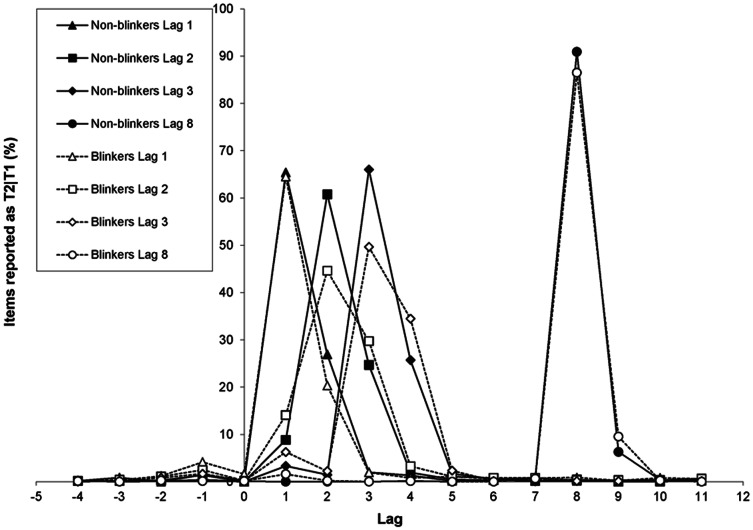
Distribution of T2|T1 reports in Experiment 1b. The percentage of letters at a particular position in the RSVP stream that were reported as T2 given correct report of T1 as a function of lag, for blinkers and non-blinkers.

A RM-ANOVA of T2|T1 revealed a significant effect of group, *F*(1,26) = 9.87, *MSE* = 21.05, *p* = .004, *η* ^2^ *_p_* = .28, whereas neither the effect of lag (*p* = .27) nor the Group × Lag interaction (*p* = .91) was significant. These findings suggest that overall, little or no suppression seemed to be present, and that the AB did not induce any suppression as a function of lag in this study. Given that many theoretical and computational models of the AB assume that the AB is caused by the suppression that is induced by T1 and/or the distractor that immediately follows T1 [14], [43], [53], [54], it is striking to find no evidence for an AB-induced suppression effect for T2, which would otherwise be reflected in a sizeable drop in performance during lags 2 and 3. However, it is important to note that because performance in the current experiment was close to ceiling, such an effect might be concealed. Figure 4 provides a more detailed picture regarding the distribution of T2|T1 reports, revealing that participants tend to report either the letter preceding or following the second target when making intrusion errors. We will discuss this pattern of intrusions further in the section below on ‘relative T2+3 intrusions’.

#### Delay

In order to measure the latency of these intrusion errors in a similar manner as [43], [44] did, we calculated the centre of mass (*C*) of reports in the window around a given target as follows: 
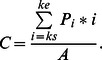



Originally employed by Chun [44], the centre of mass corresponds to the average reported serial position relative to the target. A positive centre of mass indicates that participants are more likely to report items following the target, whereas a negative centre of mass would indicate a bias to report items preceding the target. If the centre of mass is more positive for T2 than for T1, this means that selection is delayed for T2 relative to T1. Order reversals were counted as incorrect in this analysis.

**Figure 5:**
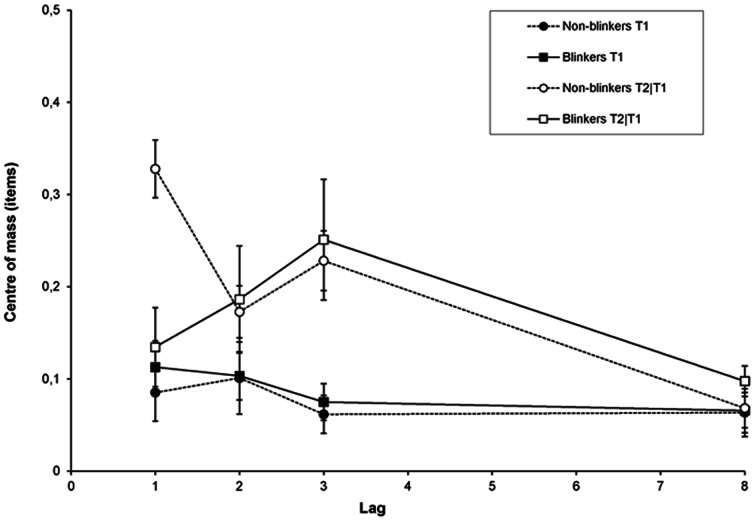
Delay in Experiment 1b. Delay of the temporal selection process expressed as the centre of mass of reports in the selection window around a given target as a function of lag, for blinkers and non-blinkers.

Figure 5 shows the measure of delay for T1 and T2 as a function of lag, for blinkers and non-blinkers. A RM-ANOVA of the centre of mass for T1 only revealed a significant main effect of lag, *F*(3, 78) = 3.03, *MSE*  = .004, *p* = .045, *η* ^2^ *_p_* = .1. For T2|T1 we found an effect of lag, *F*(2.1, 54.64) = 6.48, *MSE* = .02, *p* = .003, *η* ^2^ *_p_* = .20; no main effect of group (*p* = .35); and a Group × Lag interaction, *F*(2.1, 54.64) = 3.63, *MSE* = .02, *p* = .03, *η* ^2^ *_p_* = .12. The non-blinkers show a delay that is particularly pronounced at lag 1, whereas for blinkers the strongest delay is observed at lag 3. Independent samples t-tests revealed a significant difference between non-blinkers and blinkers at lag 1 only, *t*(26) = 3.88, *SE* = .51, *p* = .001. This might reflect a difference in the use of letters following the second target for the two groups, however, it must be noted that this could also reflect a difference in the binding of letter identity and colour, which is discussed more extensively in the general discussion.

#### Diffusion

**Figure 6:**
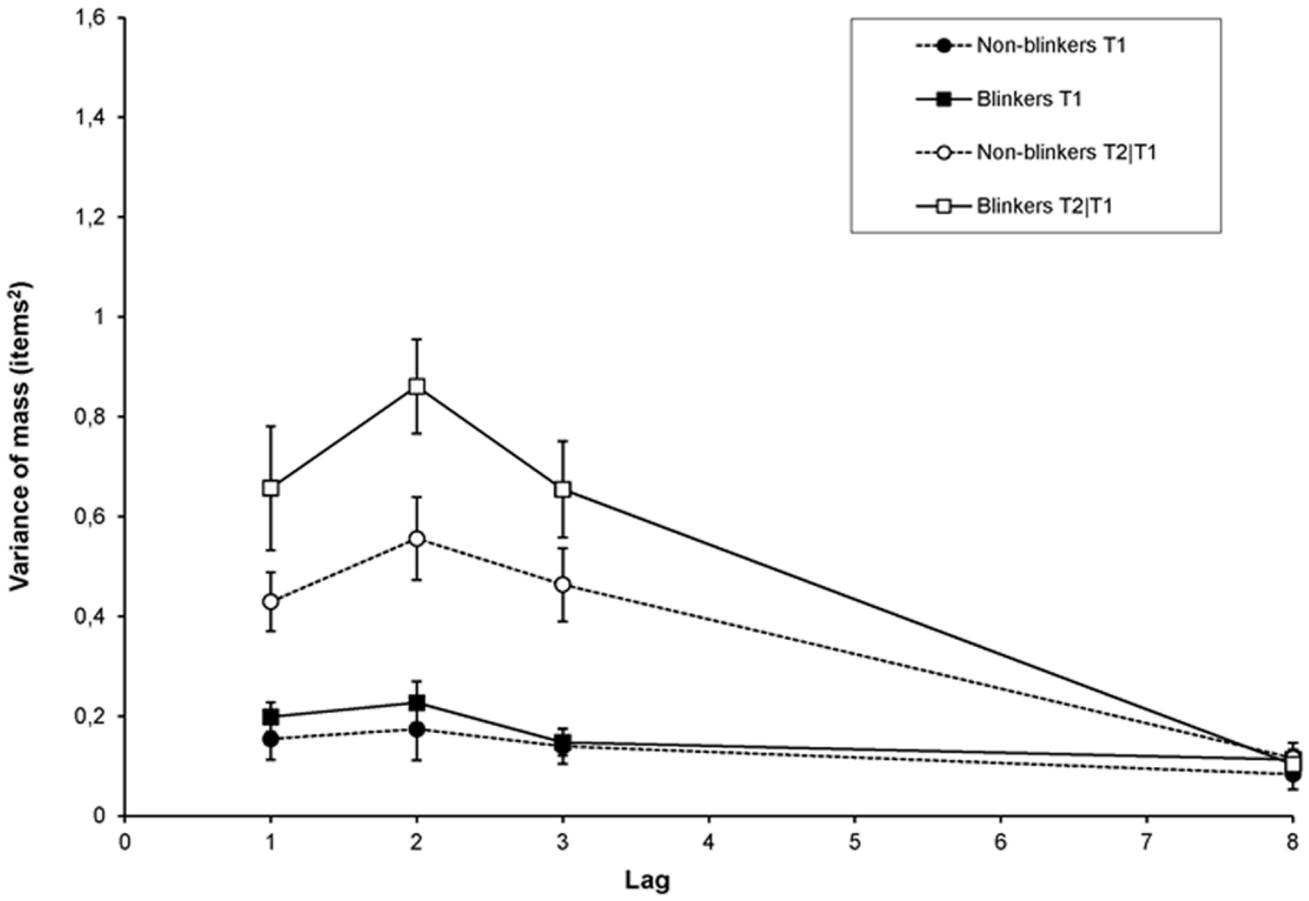
Diffusion in Experiment 1b. Diffusion of the temporal selection process expressed as the variance of the centre of mass in the selection window around T1 or T2 as a function of lag, for blinkers and non-blinkers.

Similarly to Vul et al. [43], we estimated the precision of selection around the centre of mass (see Figure 6) by calculating the variance of the centre of mass (*V*), as follows: 
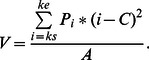
Here, the variance of the centre of mass reveals to which extent the reports of the letters are diffused around the centre of mass, reflecting the spread of selection. Again, order reversals were counted as incorrect.

For T1, we only found a significant effect of lag, *F*(1.82, 47.36) = 6.41, *MSE* = .01, *p* = .004, *η* ^2^ *_p_* = .2; whereas for T2|T1 we found a significant effect of group, *F*(1,26) = 4.29, *MSE* = .21, *p* = .048, *η* ^2^ *_p_* = .14; and lag, *F*(2.2, 57.2) = 33.01, *MSE* = .08, *p*<.001, *η* ^2^ *_p_* = .56; but no significant Group × Lag interaction (*p* = .11). These results clearly reflect that—compared to non-blinkers—blinkers are less precise in selecting the second but not the first target.

#### Relative T2+3 intrusions

The relatively high performance within the 7-item window reveals that response errors were far from random, as illustrated in Figures 3 and 4. The latter figure indicates that for lags 2 and 3, blinkers show more post-target intrusions than non-blinkers do. However, blinkers show more errors overall, so a more meaningful comparison would be to determine the pattern of relative intrusion errors, controlling for differences in the total error rate. To that end, we examined the percentage of erroneously selected letters presented at one to three serial positions following a target, relative to all errors on a given lag. Order reversals were counted as incorrect. For T1, as well as for T2 at lag 8, the number of post-target intrusions was insufficient to allow for a meaningful analysis. Therefore, this analysis was restricted to T2|T1 at lags 1 to 3 only. For this analysis, the average number of trials over participants available in blinkers was 16.3, 25.3, and 27.0 for lags 1, 2, and 3, respectively. In non-blinkers this was 21.2, 19.8, and 20.4 for lags 1, 2, and 3, respectively.

**Figure 7:**
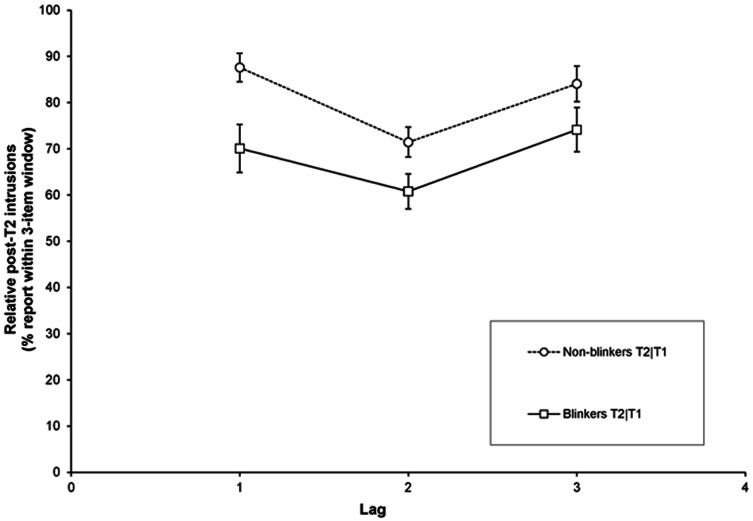
Intrusion errors in Experiment 1b. Percentage of erroneously selected letters (relative to all trials with an incorrect T2 response) presented 1–3 serial positions following T2 as a function of lag, for blinkers and non-blinkers.

In Figure 7 the percentage T2+3 intrusions relative to all errors on a given trial are plotted as a function of lag. A RM-ANOVA of the T2+3 intrusions with lag (1, 2, and 3) as a within-subjects factor and group (non-blinkers and blinkers) as a between-subjects factor revealed significant effects for lag, *F*(1.46, 37.91) = 7.97, *MSE* = 264.53, *p* = .003, *η* ^2^ *_p_* = .24; and group, *F*(1,26) = 9.93, *MSE* = 339.4, *p* = .004, *η* ^2^ *_p_* = .28; but a significant Group × Lag interaction was not found (*p* = .48). Thus, compared to blinkers, when a selection error was made, the T2 response of non-blinkers more frequently matched one of the items following the second target. In contrast to the pattern of absolute intrusion rates (see Figure 4), the current analysis of relative post-target intrusions shows that this was not only the case at lag 1, but also at lags 2 and 3 (see Figure 7).

#### Order reversals

The percentage of order reversals for trials during which T1 and T2 were both correct was 8.3%, .3%, .5%, and .2% at lags 1, 2, 3, and 8, respectively. A significant main effect of lag reflected the decrease of order reversals as a function of lag, *F*(1.1, 28.39) = 23.83, *MSE* = 49.52, *p*<.001, *η* ^2^ *_p_* = .48. No effect of group (*p* = .6) or an interaction effect between group and lag (*p* = .54) was found, suggesting no difference in order reversals between non-blinkers and blinkers. Given that AB magnitude was larger in Experiment 1b than in Experiment 1a for both groups, it is perhaps surprising that there were substantially more order reversals in Experiment 1a. An explanation might at least partially lie in the fact that the SOA was much shorter in Experiment 1a (80 ms) than in Experiment 1b (120 ms).

### Experiment 2

After initial analysis, 21 students were excluded from further analyses due to insufficient identification performance of T1 (<70%). In total, 111 participants remained for further analyses. Given that Experiment 2 featured a wide range of AB magnitudes, we treated AB magnitude in the analyses of Experiment 2 as a continuous variable. However, for the sake of clarity, figures for Experiment 2 feature three subgroups, based on individuals' AB magnitude in the first block of the experiment. Mean AB magnitude was 15.9% (range = 1.3–27.0%) for the group of ‘small blinkers’, 39.1% (range = 27.0–47.5%) for the group of ‘medium blinkers’, and 60.3% (range = 48.2–92.8%) for the group of ‘large blinkers’.

**Figure 8:**
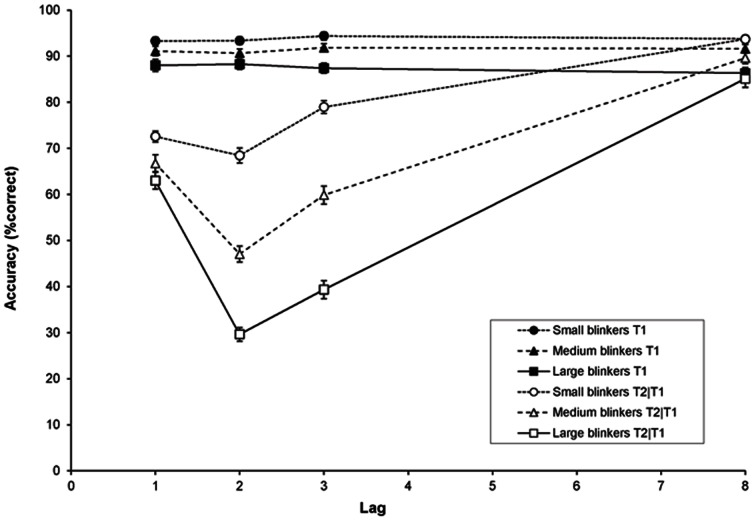
Target accuracy in Experiment 2. Mean percentage correct report of T1 (black symbols) and T2 given correct report of T1 (white symbols) as a function of lag, for small blinkers (circles), medium blinkers (triangles), and large blinkers (squares). Error bars reflect standard error of the mean.

In Figure 8, T1 accuracy and T2|T1 accuracy are plotted as a function of lag (1, 2, 3, and 8), for the small blinkers (circle symbols), the medium blinkers (triangle symbols), and the large blinkers (square symbols). A RM-ANCOVA of T1 performance with lag (1, 2, 3, and 8) as a within-subjects factor and AB magnitude as a continuous between-subjects factor (i.e., covariate) revealed no effect of lag (*p* = .07), but there was a main effect of AB magnitude, *F*(1, 109) = 22.37, *MSE* = 116.0, *p*<.001, *η* ^2^ *_p_* = .17, and a significant AB magnitude × Lag interaction, *F*(3, 327) = 3.3, *MSE* = 10.63, *p* = .022, *η* ^2^ *_p_* = .03.

A RM-ANCOVA of T2|T1 revealed an effect of lag, *F*(3, 327) = 40.93, *MSE* = 63.83, *p*<.001, *η* ^2^ *_p_* = .27; AB magnitude, *F*(1, 109) = 365.59, *MSE* = 134.9, *p*<.001, *η* ^2^ *_p_* = .77; and a significant AB magnitude × Lag interaction, *F*(3, 327) = 90.39, *MSE* = 63.83, *p*<. 001, *η* ^2^ *_p_* = .45. These results confirm the presence of clear individual differences in AB magnitude, as illustrated in Figure 8.

#### Suppression

The amount of suppression was calculated in the same manner as in Experiment 1b. Again, a paired t-test revealed that the accuracy within the 7-item window differed significantly from the level of chance, *t*(110) = 61.47, *SE* = .82, *p*<.001; *t* (110) = 99.74, *SE* = .52, *p*<.001; *t*(110) = 95.43, *SE* = .56, *p*<.001; *t*(110) = 199.97, *SE* = .56, *p*<.001 for lags 1, 2, 3, and 8, respectively.

**Figure 9:**
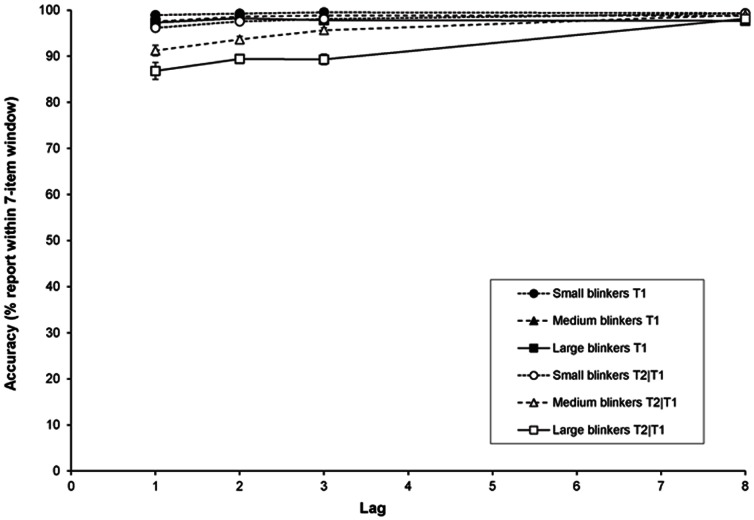
Suppression in Experiment 2. Suppression of the temporal selection process expressed as the accuracy of reporting an item within the 7-item window around a given target as a function of lag, for small, medium, and large blinkers.

Figure 9 shows the accuracy within a 7-item window for T1 and T2|T1 as a function of lag, for the different groups. A RM-ANCOVA of T1 showed an effect of AB magnitude, *F*(1, 109) = 23.42, *MSE* = 6.45, *p*<.001, *η* ^2^ *_p_* = .18; but no significant effect of lag (*p* = .45) or an AB magnitude × Lag interaction (*p* = .45).

A RM-ANCOVA of T2|T1 revealed no effect of lag (*p* = .30), but there was an effect of AB magnitude, *F*(1, 109) = 88.29, *MSE* = 46.76, *p*<.001, *η* ^2^ *_p_* = .45; and an AB magnitude × Lag interaction, *F*( 1.94, 211.65) = 14.81, *MSE* = 29.47, *p*<.001, *η* ^2^ *_p_* = .12. Thus, as can be seen in Figure 9, little or no suppression occurred in small blinkers, whereas suppression of distractors as a function of lag clearly occurred in large blinkers. However it must be noted that, as in Experiment 1b, the ceiling effect might be a restrictive factor here.

**Figure 10:**
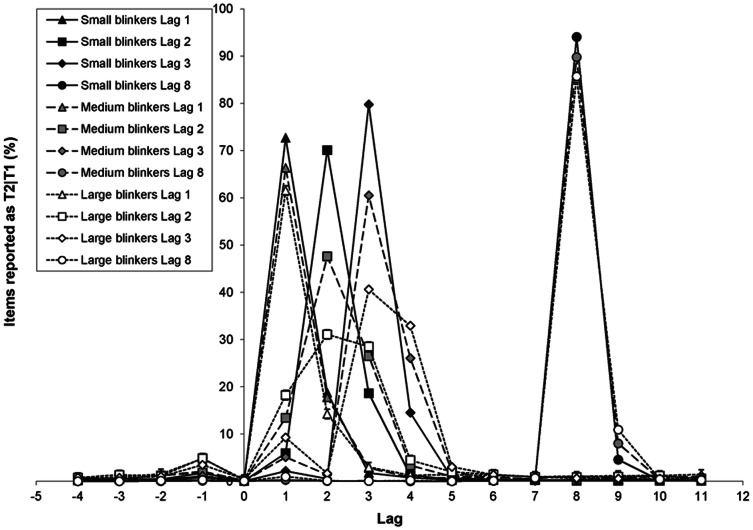
Distribution of T2|T1 reports in Experiment 2. The percentage of letters at a particular position in the RSVP stream that were reported as T2 given correct report of T1 as a function of lag, for small, medium, and large blinkers.

The distribution of T2|T1 reports can be found in Figure 10. Here it can be seen that, again, the main contributors of the high accuracy in the 7-item window are the reports of the targets either preceding or following the target, plus the reports of the target itself.

#### Delay

**Figure 11:**
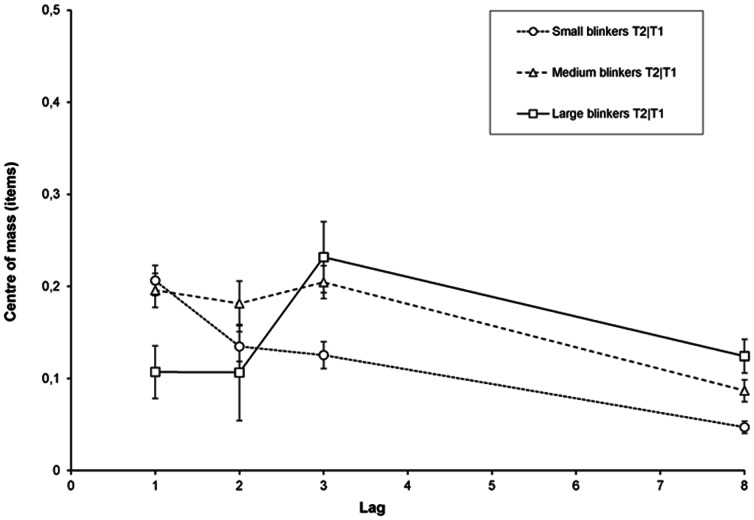
Delay in Experiment 2. Delay of the temporal selection process expressed as the centre of mass of reports in the selection window around a given target as a function of lag, for small, medium, and large blinkers.

The amount of delay during the temporal selection process was calculated as in Experiment 1b. The results for T2|T1 as a function of lag are plotted in Figure 11. For the sake of clarity, T1 is not plotted. A RM-ANCOVA of T1 showed an effect of lag, *F*(3, 327) = 4.18, *MSE* = .003, *p* = .006, *η* ^2^ *_p_* = .04; and AB magnitude, *F*(1, 109) = 7.99, *MSE* = .02, *p* = .006, *η* ^2^ *_p_* = .07; but no significant AB magnitude × Lag interaction was found (*p* = .66).

For T2|T1, a RM-ANCOVA showed an effect of lag, *F*(3, 327) = 12.81, *MSE* = .02, *p*<.001, *η* ^2^ *_p_* = .11; no main effect of AB magnitude (*p* = .33); but a significant AB magnitude × Lag interaction, *F*(3, 327) = 12.54, *MSE* = .02, *p*<.001, *η* ^2^ *_p_* = .10. As shown in Figure 11, consistent with our findings in Experiment 1b, there was a remarkable delay at lag 1 for small blinkers, whereas for large blinkers the delay was most pronounced at lag 3.

#### Diffusion

**Figure 12:**
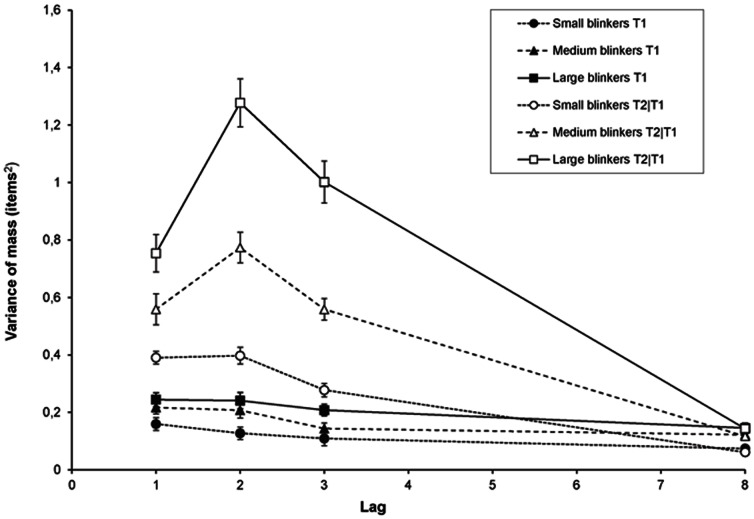
Diffusion in Experiment 2. Diffusion of the temporal selection process expressed as the variance of the centre of mass in the selection window around T1 or T2 as a function of lag, for small, medium, and large blinkers.

Shown in Figure 12, diffusion during the temporal selection process was calculated as in Experiment 1b. A RM-ANCOVA of T1 revealed a main effect of AB magnitude, *F*(1, 109) = 7.55, *MSE* = .09, p = .007, *η* ^2^ *_p_* = .07; but no significant effect was found of lag (*p* = .24) or AB magnitude × Lag interaction (*p* = .76).

For T2|T1 we found a significant effect of lag, *F*(3, 327) = 9.64, *MSE* = .05, *p*<.001, *η* ^2^ *_p_* = .08; AB magnitude, *F*(1, 109) = 164.85, *MSE* = .16, *p*<.001, *η* ^2^ *_p_* = .60; and also an AB magnitude × Lag interaction, *F*(3, 327) = 60.16, *MSE* = .05, *p*<.001, *η* ^2^ *_p_* = .36. These results clearly confirm the results of Experiment 1b, namely that the temporal selection process of small blinkers is more precise than that of large blinkers. The significant interaction with lag as observed in the current experiment indicates that this is especially the case during the AB interval.

#### Relative T2+3 intrusions

**Figure 13:**
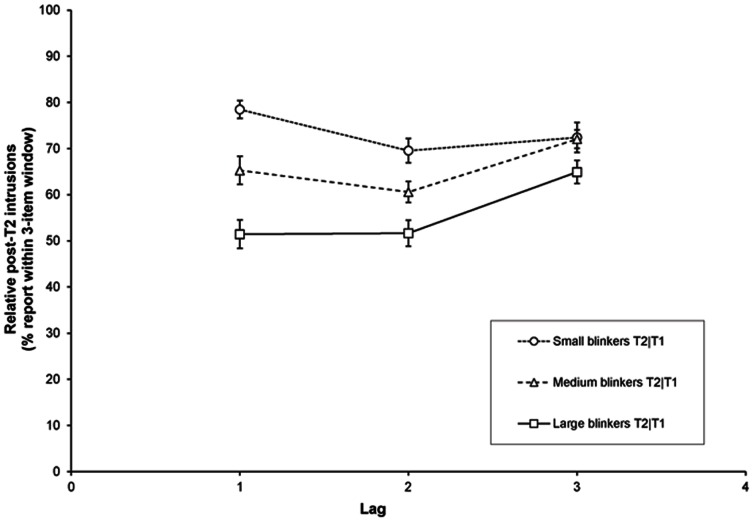
Intrusion errors in Experiment 2. Percentage of erroneously selected letters (relative to all trials with an incorrect T2 response) presented 1–3 serial positions following T2 as a function of lag, for small, medium, and large blinkers.

Focusing on lags 1 to 3, we examined the percentage of erroneously selected letters presented one to three serial positions following T2 relative to all errors on a given lag, as shown in Figure 13. For this analysis, the average number of trials over participants available was 14.4, 21.1, and 19.9 for lags 1, 2, and 3, respectively.

A RM-ANCOVA revealed a significant effect of lag, *F*(2, 218) = 4.6, *MSE* = 177.7, *p* = .011, *η* ^2^ *_p_* = .04; AB magnitude, *F*(1, 109) = 61.52, *MSE* = 368.31, *p*<.001, *η* ^2^ *_p_* = .36; and AB magnitude × Lag, *F*(2, 218) = 10.09, *MSE* = 177.7, *p*<.001, *η* ^2^ *_p_* = .09, such that small blinkers made relatively more post-target intrusions than large blinkers did, particularly at the shorter lags (see Figure 13). Thus, besides making fewer mistakes, small blinkers made more educated guesses with the T2 response frequently matching with one of the subsequent items in the RSVP stream.

#### Order reversals

As in the former experiments, we calculated the percentage of order reversals for trials during which T1 and T2 were both reported correctly. Here, we found no effect of lag (*p* = .065), but there was a significant effect of AB magnitude, *F*(1, 109) = 24.38, *MSE* = 15.18, *p*<.001, *η* ^2^ *_p_* = .18; and a significant AB magnitude × Lag interaction, *F*(1.14, 124.34) = 19.09, *MSE* = 10.8, *p*<.001, *η* ^2^ *_p_* = .15, such that large blinkers had more order reversals than small blinkers did, particularly at the short lags. These results suggest that a small or absent AB does not come at a cost for temporal order information, and is better preserved for small blinkers than for large blinkers.

## General Discussion

The aim of this study was threefold. Previously, we found that some individuals show little or no AB when required to identify two target letters presented in a sequential stream of non-target digits. Our first goal was to investigate whether these ‘non-blinkers’ would continue to show no AB when required to identify two red target letters amongst a stream of black non-target letters, thus testing the generality of their remarkable ability in avoiding an AB. Earlier, it was found that they failed to do so when targets had to be selected based on rotation or semantic features [16], [20]. After replicating the differential performance between blinkers and non-blinkers in a standard alphanumeric AB task, we found that when targets and distractors could only be distinguished on the basis of colour, a substantial AB occurred in both groups. Though colour is a stimulus property that is available relatively early in the processing pathway [40]–[42], apparently early target selection was not possible to the extent that non-blinkers failed to avoid the occurrence of an AB. Combined with the previous observation of an AB in non-blinkers when alphanumeric stimuli were presented in the auditory modality [18], the current results seem to suggest that the non-blinkers' ability might indeed be quite task-specific, requiring the presence of visual alphanumeric category information. However, given that AB magnitude in our coloured targets task remained smaller in non-blinkers than in blinkers, there must be more to the story.

Interestingly, the coloured targets paradigm as employed here allowed us to study individual differences in target selection efficiency in more detail. More specifically, our second aim was to study possible differences in the temporal profile of blinkers and non-blinkers by examining the amount of suppression, delay, and diffusion of the temporal selection process during the AB [43]. We expected to find differences in these three dissociable dimensions of temporal selection, because even in the coloured target task clear differences in AB magnitude were observed.

### Suppression

Surprisingly, little suppression was observed in both Experiments 1b and 2; the efficacy of selection, measured as the percentage of trials during which an item was reported from a 7-item window around either T1 or T2 (i.e., spanning three items before to three items after the target), was generally high. In Experiment 1b, a significant difference between blinkers and non-blinkers in the amount of suppression for T2 was found, which, however, was not modulated by lag. This finding is similar to what was reported by Popple and Levi [49]. It must be noted though that in their study, as well as in the current one, patterns of AB-induced suppression may have been obscured by ceiling effects.

In Experiment 2, employing a larger sample of subjects and thus a wider range of AB magnitudes, the interaction of AB magnitude and lag reflected signs of suppression of T2 and the surrounding distractors at the shortest lags for large blinkers, whereas small blinkers continued to show no suppression whatsoever. Although the finding of suppression as a function of lag corresponds with findings from previous studies [43], [44], [50], all of these papers reported substantially more suppression.

An explanation for these differential findings might lie in differences in methods, stimuli, and overall task difficulty. Whereas both our study and that of Popple and Levi [49] employed integral dimensions of the stimuli as the relevant features (colour and shape), Vul et al. [43] as well as Chun [44] used composed targets (a letter surrounded by an annulus or coloured frame). Although the study by Botella and colleagues [50] did use colour as an integrated target feature, they introduced a task-switch by varying the colour of the two targets, and possibly reduced the effectiveness of colour as a target-specific feature by also varying the colour of each distractor in the stream. It is thus not inconceivable that the latter studies introduced additional factors into the AB task that further complicated the binding and subsequent selection of targets. In addition, the level of overall performance in [43] was dramatically low (∼10–50%), making comparisons with other AB studies—that typically feature much higher performance—difficult.

Another notable finding pertains to the individual differences in the amount of suppression. In multiple studies it has been suggested that the AB is due to a failure to effectively suppress distractors [14], [46], [47], [52]. Specifically, based on findings in their priming study, Dux and Marois [14] suggested that large blinkers in particular fail to suppress the processing of irrelevant distractors, whereas small blinkers frequently manage to avoid an AB by successful suppression of these distractors. If that would indeed be the case, however, one would expect to see strong suppression in non-blinkers and little or no suppression in large blinkers, exactly opposite to the pattern of findings reported here.

Instead, we propose that non-blinkers are somehow able to select targets at an earlier processing stage than blinkers do, to some extent even when targets are not defined by alphanumeric category. Consequently, compared to blinkers, non-blinkers may have little need to suppress distractors, as stable target representations can more readily and easily be formed. The less effective this early selection, the stronger the need for suppression at a later stage of processing, a pattern that is indeed in line with the levels of suppression that we observed in small, medium, and large blinkers, respectively (see Figure 9). However, it must be noted that given the relatively modest amount of suppression observed in the current study, it is hard to conceive that suppression alone can account for the significant AB that was obtained in the majority of participants. Moreover, it remains puzzling why the strongest suppression tended to occur at lag 1, whereas the strongest AB was consistently found at lag 2.

### Delay

Another surprising finding emerged in the latency measure of the intrusion errors. Following Vul et al. [43] and Chun et al. [44], the centre of mass was calculated as a measure of delay. Whereas for large blinkers, the maximal delay was consistently found at lag 3, for small blinkers the maximum in both experiment 1b and 2 was observed at lag 1. This latter finding, however, may at least partly reflect an artefact of the T2 centre of mass calculation, and at first sight does not seem to be very meaningful. That is, the small blinkers' seemingly large delay at lag 1 may be the simple consequence of a) the fact that the diffusion of responses was substantially smaller for small blinkers than for large blinkers (who made intrusions from a wider window; see section below), b) the fact that small blinkers made relatively more post-target intrusions than blinkers did (see Figures 7 and 13), and c) the fact that correct T1 responses are excluded from the calculation. The combination of these factors at lag 1 may thus be responsible for an inflated centre of mass for small blinkers, and a centre of mass that is close to zero for large blinkers. However, given that the results found here correspond to the pattern of relative post-target intrusion errors (further discussed below), they may nevertheless reflect a genuine difference between small and large blinkers.

The pattern of results is quite different from that reported by Vul et al. [43] and Chun [44], who both reported finding a negative centre of mass at the shortest lags. Again, an explanation might lie in differences in methods, stimuli, and overall task difficulty, as well as the fact that their participants showed more suppression than the individuals in the current study did.

### Diffusion

Perhaps the most telling and straightforward finding is provided by the measure of diffusion, expressing the precision of selection for each group of individuals. Calculated as the variance of the centre of mass, the amount of diffusion showed a consistent pattern that matched closely with that of the AB, reaching the lowest temporal precision at lag 2. Although the amount of overall diffusion was much lower than that reported by Vul et al. [43], the pattern of diffusion as a function of time between the targets is very similar. In addition, our current findings clearly showed that, compared to small blinkers, large blinkers were less precise in selecting the second but not the first target.

This pattern of diffusion fits with the idea that non-blinkers are able to select targets at an earlier processing stage than blinkers do. Early target selection may reduce interference from distractors, allowing subsequent processing of the targets to proceed faster and more accurately in non-blinkers than in blinkers, reflected in earlier P3s [3], [20] and less diffusion.

### Relative intrusion errors

In addition to these three dimensions of temporal selection, we analysed the percentage of erroneously selected letters presented one to three serial positions following T2 relative to all errors on a given lag (see Figures 7 and 13). Errors in the temporal selection process have been studied before [44], [49], [50], but individual differences were not considered and differences in the total number of errors were not controlled for. Given that intrusions of items following T2 are inherently related to the total number of errors made, we studied the relative number of intrusions, allowing comparisons between blinkers and non-blinkers in the type of intrusions irrespective of the total rate of response errors. In both Experiments 1b and 2, we found that non-blinkers and small blinkers made relatively more post-T2 intrusions than blinkers did. In Experiment 2, within the group of small blinkers, most post-T2 intrusions were made at lag 1, whereas within the group of large blinkers most of these intrusions occurred at lag 3. This pattern matches quite well with the differences in delay that we observed for the different groups, but poses a challenge in terms of interpretation. Although we argued that the latter differences might at least partly be due to the way in which the centre of mass was calculated, the significant interaction between group and lag in the relative post-T2 intrusions does indicate systematic differences in the selection process employed by blinkers and non-blinkers, especially at lag 1.

Note however, that some caution is generally required in the interpretation of what a shift in the centre of mass as well as the number of relative post-target intrusion errors actually reflect. Given that the particular task employed in the current study required the binding of a colour to a particular letter, the delay that is associated with a positive shift in the centre of mass or an increase in post-target intrusions may be due to non-blinkers and blinkers having differential processing speeds in either the colour, letter, or the binding of features (or a combination thereof). Future research is needed to isolate these different components of the temporal selection process.

### Relative order reversals

In response to the proposition that the AB reflects a cognitive strategy of enforcing an episodic distinction between successive stimuli of Wyble, Bowman, and Nieuwenstein [29], our third and final aim was to determine whether avoiding an AB comes at a cost. Given the non-blinkers' ability to largely avoid the occurrence of an AB, information concerning temporal order and the correct binding of features into targets might be compromised in non-blinkers. If that were indeed the case, non-blinkers should show relatively more order reversals, compared to large blinkers. However, while correcting for differences in target accuracy, the opposite pattern of results was observed. Although no significant difference in relative order reversals was found between blinkers and non-blinkers in Experiment 1b, individuals with little or no AB showed fewer rather than more order reversals than large blinkers as showed in Experiment 1a and 2. Even though the AB may have a functional role in providing episodic distinctiveness, our results suggest that avoiding an AB does not come at a cost for temporal order information.

## Conclusions

By studying individual differences in response errors, we found that only a modest amount of suppression of T2 and surrounding distractors was present in blinkers. In addition, lower accuracy was closely accompanied by reduced precision during target selection in blinkers. In comparison, the temporal selection process seems to be faster and more precise in non-blinkers, and we found no evidence of suppression. Non-blinkers did show a sizeable AB when target selection was based on colour features rather than alphanumeric category, but continued to outperform blinkers. Finally, we found that non-blinkers did not lack episodic distinctiveness; temporal order information was actually preserved better in individuals with a small rather than a large AB. Intriguingly, non-blinkers showed most intrusions as well as a selection delay at lag 1, a finding that deserves further investigation.
