# Correction: Individual Differences in the Attentional Blink: The Temporal Profile of Blinkers and Non-Blinkers

**DOI:** 10.1371/annotation/222971f5-0ce8-4776-943d-b2854a3836eb

**Published:** 2013-12-05

**Authors:** Charlotte Willems, Stefan M. Wierda, Eva van Viegen, Sander Martens

This article has been republished to correct equation formatting errors that were introduced during the production process. Please download the PDF again for the correct version. 

